# The EUROPEP questionnaire for patient’s evaluation of general practice care: Bulgarian experience

**DOI:** 10.3325/cmj.2017.58.63

**Published:** 2017-02

**Authors:** Rositsa Dimova, Rumyana Stoyanova, Donka Keskinova

**Affiliations:** 1 Medical University of Plovdiv, Faculty of Public Health, Department of Healthcare Management and Health Economics, Plovdiv, Bulgaria; 2 Medical University of Plovdiv, Department of Health Management and Health Economics, Plovdiv, Bulgaria; 3 Plovdiv University “Paisii Hilendarski”, Applied and Institutional Sociology, Plovdiv, Bulgaria

## Abstract

**Aim:**

To validate the Bulgarian EUROPEP-questionnaire and its implementation to measure patient evaluation of general practice care in Bulgarian population.

**Methods:**

A multicenter cross-sectional study was conducted at twenty five primary care practices from South-Central Region of Bulgaria. A total of 1000 adult patients aged over 18 years and visiting the practice for more than a year were approached consecutively to take part in the study. The internal consistency and test-retest reliability of the EUROPEP questionnaire were evaluated. To confirm the construct validity of the questionniare, еxplanatory factor analysis was performed.

**Results:**

Cronbach’alpha for “clinical behaviour” is 0.95 and for “organisation of care” 0.81. Factor analysis identifed two factors, which accounted for 77.0% of the total variation in these items. On average, 58.7% of respondents rated the level of care received as excellent. The waiting time in the waiting room was the item most poorly rated (33.8%). The item “keeping patients' records and data confidential” was the most highly rated (88.8%). Patients were less satisfied with “providing quick services for urgent health problems” (78.5% excellent or good) and “getting an appropriate for them appointment” (76.2% excellent or good).

**Conclusion:**

Two scales with satisfactory psychometric properties were established in the Bulgarian version of the EUROPEP-questionnaire. The study identified areas requiring improvement in general practice, such as reduction in waiting times and obtaining patient’s convenience appointment.

Health care systems based on person-centered care are designed to respect patient expectations, needs, and priorities (1). Patient perceptions of the quality of health care services are increasingly recognized as relevant to the evaluation of health care outcomes (2). Patient satisfaction, which is generally a multidimensional construct, has thus become a valuable indicator of medical care quality (2,3). Review of the available literature shows that patient satisfaction is related to general practitioners’ good communication skills and establishment of a good patient-physician relationship (4,5). In general, improved patient satisfaction with health care contributes to patient compliance with treatment and improves health outcomes (4,5).

There is no universal gold standard for measuring patient satisfaction. Different surveys of patient satisfaction with general practice care in Europe used the European Task Force on Patient Evaluations of General Practice Care (EUROPEP) questionnaire. It is an internationally standardized and validated instrument using patient evaluation of their regular general practitioner (GP) based on their experience over the preceding year (6). Initially, the EUROPEP-instrument was developed to allow the comparison of outcomes in general practice care across Europe and provide an educational feedback to both general practitioners and patients (6,7).

In Bulgaria, after the health care reform in 2000, various surveys of patient satisfaction have been performed. Regardless of the accumulated data on patient satisfaction with general practice care, there has been no instrument allowing for the comparison of the results with those from other studies. As Bulgaria was not included in the initial comparative study of patient satisfaction in European countries, a special survey using the Bulgarian EUROPEP-instrument was conducted to collect the data on patient satisfaction with general practice. To our knowledge, this is the first study of patient satisfaction in which the Bulgarian EUROPEP-questionnaire was used.

The aim of the study was the validation of Bulgarian EUROPEP-questionnaire and its implementation to measure patient evaluation of general practice care in Bulgarian population.

## PARTICIPANTS AND METHOD

We performed the translation and validation of Bulgarian EUROPEP-instrument and carried out the first cross-sectional study of patient evaluation of general practice care using the Bulgarian EUROPEP-instrument as a part of the Medical University of Plovdiv project to develop a standardized methodology for large-scale measurement of patient experiences with general practitioners in Bulgaria. The study was conducted in a randomly selected region of Bulgaria among 1000 adult general practice patients from April 2015 to July 2015.

### EUROPEP instrument – translation and validation of Bulgarian version

The EUROPEP-instrument is a questionnaire that includes 23 items categorized into five qualitative domains each measuring different aspects of care including doctor-patient-relationship; medical care; information and support; continuity and co-operation, and accessibility. All items are aggregated into two dimensions: clinical behavior (items 1-16) and organization of care (items 17-23) (Table 1).

**Table 1:** Test-retest reliability of the Bulgarian translation of EUROPEP-instrument evaluated in 160 patients*

Bulgarian EUROPEP-instrument subscales	Administration of questionnaire					
	Mean І measurement	Mean І І measurement	Wilcoxon test	Spearman-Brown coefficient (rsb)	Cronbach’s α	Inter-item correlation
Relation and communication					0.884	0.559
1. Making you feel you had time during consultation?	4.28	4.25	0.65^†^	0.774		
2. Interest in your personal situation?	4.32	4.29	0.84^†^	0.820		
3. Making it easy for you to tell him or her about your problem?	4.20	4.12	1.82^†^	0.809		
4. Involving you in decisions about your medical care?	4.22	4.27	1.15^†^	0.804		
5. Listening to you?	4.40	4.45	1.11^†^	0.752		
6. Keeping your records and data confidential?	4.63	4.54	1.77^†^	0.687		
Medical care					0.889	0.628
7. Quick relief of your symptoms?	4.23	4.27	0.82^†^	0.815		
8. Helping you to feel well so that you can perform your normal daily activities?	4.23	4.25	0.47^†^	0.797		
9. Thoroughness?	4.18	4.16	0.57^†^	0.803		
10. Physical examination of you?	4.21	4.12	2.06	0.738		
11. Offering you services for preventing diseases (eg, screening, health checks, immunizations)	4.02	4.04	0.74^†^	0.784		
Information and support					0.922	0.751
12. Explaining the purpose of tests and treatments?	4.08	4.07	0.18^†^	0.824		
13. Telling you what you wanted to know about your symptoms and/or illness?	4.20	4.19	0.18^†^	0.772		
14. Helping you deal with emotional problems related to your health status?	4.00	4.00	0.15^†^	0.835		
15. Helping you understand the importance of following his or her advice	4.03	4.03	0.31^†^	0.804		
Continuity and co-operation					0.892	0.805
16. Knowing what s/he had done or told you during consultation?	4.11	4.06	0.74^†^	0.812		
17. Preparing you for what to expect from specialist or hospital care?	4.02	3.95	1.12^†^	0.797		
Availability and accessibility					0.826	0.439
18. The helpfulness of the staff (other than the doctor)?	3.94	3.85	1.82^†^	0.742		
19. Getting an appointment to suit you?	3.70	3.81	1.94^†^	0.790		
20. Getting through to the practice on the telephone?	4.02	3.95	1.12^†^	0.845		
21. Being able to speak to the general practitioner on the telephone?	4.21	4.20	1.28^†^	0.885		
22. Waiting time in the waiting room?	3.41	3.39	0.30^†^	0.803		
23. Providing quick services for urgent health problems?	3.95	3.90	0.85^†^	0.771		

* The questions in the table are the back translation of Bulgarian to English language.

† *P* > 0.05

**Figure 1:**
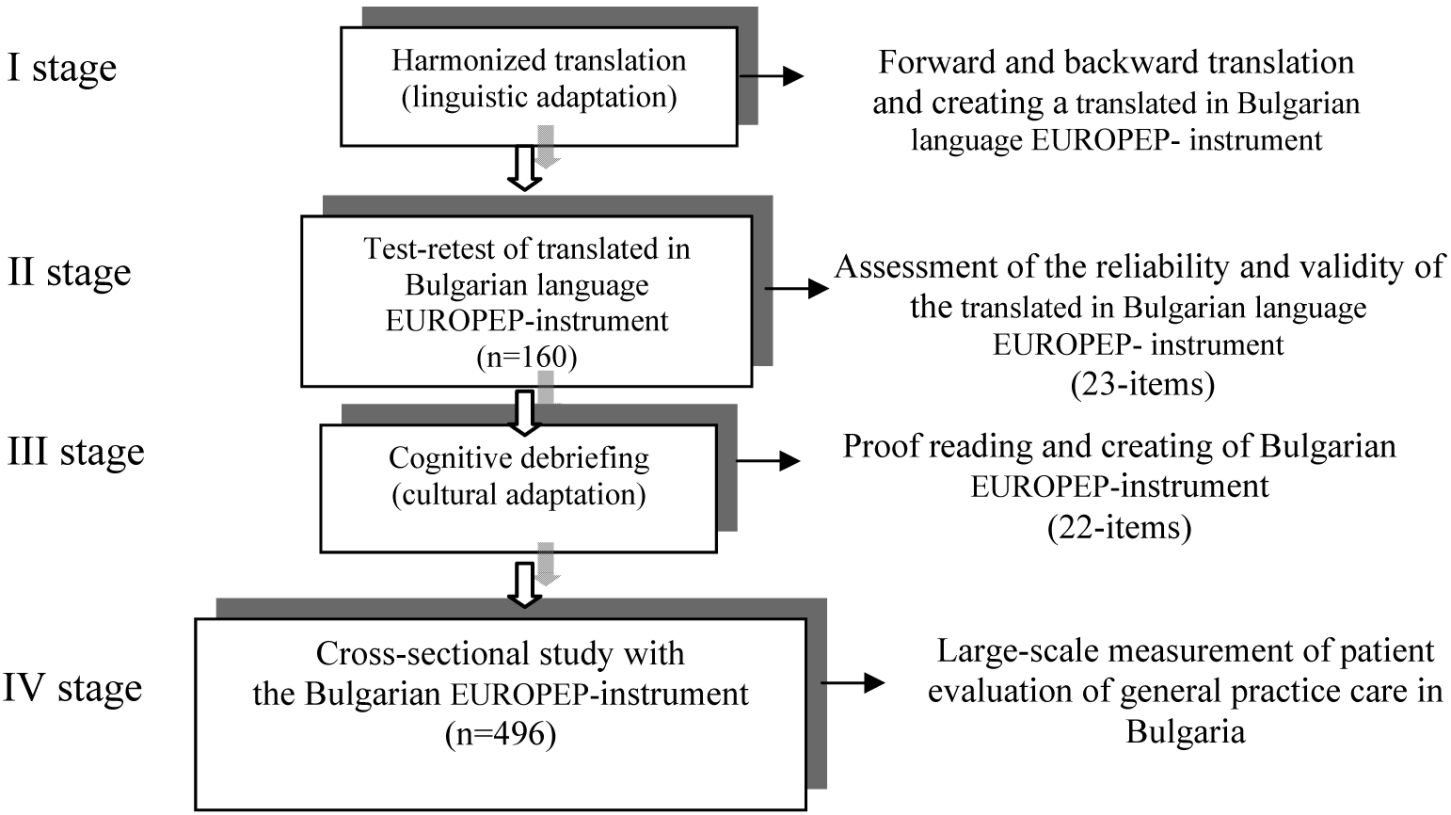
Stages in the validation process of the translated Bulgarian EUROPEP-version and subsequent large-scale measurement of patient’s evaluation of general practice care.

Responses to each item are rated on a five-point Likert scale (from 1 = poor to 5 = excellent). The EUROPEP-instrument was linguistically validated according to a standard procedure (8) and cross-culturally adapted (9) into Bulgarian in several stages (Figure 1).

*Stage І.* English source version of the EUROPEP questionnaire was translated into Bulgarian (forward translation) by three independent translators, who provided written rationales for decision making, linguistic difficulties, and problems regarding the content. The three Bulgarian versions were compared and synthesized at a consensus meeting and the subsequent consensus translation was back-translated into English by a professional translator who had no access to the original English version of the questionnaire. Furthermore, questions on patient age, gender, educational level, place of residence, perceived health status, employment status, presence of chronic diseases, and number of GP appointments over the previous year were incorporated as factorial variables. The final outcome was the adapted Bulgarian version of the EUROPEP-instrument.

*Stage ІІ.* The psychometric quality of Bulgarian EUROPEP-instrument was tested on a convenience sample of 160 patients. We selected 8 general primary practices in the city of Plovdiv and asked each of the general physicians to give 20 of their patients a copy of the questionnaire and a cover letter. Patients were eligible for participation if they were aged 18 years or more, had a valid health insurance, had been registered with the same GP practice continuously for at least one year before the date of sample selection, and they had visited their GP at least once in that period. After providing informed consent, the patients completed the questionnaire at home and sent it by post to the Department of Health Management and Health Economics, Medical University of Plovdiv. After the filled-out questionnaire was received, it was sent again to the same patients four weeks later. The questionnaire had to be completed twice by the same patients, four weeks apart, to test the reliability of their answers.

*Stage ІІІ*. Cognitive interviews were performed using concurrent think aloud and probing techniques, as described elsewhere (10), to elicit information about potential problems in the Bulgarian translation of the EUROPEP-instrument. At the cognitive debriefing, the translated Bulgarian EUROPEP-instrument was administered to 7 patients, who met the specified age and other representative criteria for the instrument target population and had no previous information about the questionnaire. The local Medical University of Plovdiv project manager, who is a sociologist, several members of the project team, and project partners (psychologist, general practitioner, and social worker) were also present. Each patient, after having completed the questionnaire, was interviewed by the local project manager. Interviews addressed each item of the Bulgarian EUROPEP-instrument if patients had indicated difficulty in understanding the question or would phrase it differently. Patients were allowed to propose alternative translation of the relevant items, which they felt were difficult to understand. Based on the suggestions or interpretations evaluated for conceptual equivalence and equivalence in construct operationalization and discussed discrepancies with the original questionnaire, the final Bulgarian EUROPEP-instrument was created.

*Stage ІV.* A cross-sectional study of patient satisfaction with general practice care was conducted using the Bulgarian EUROPEP questionnaire. The preliminary results of the Bulgarian EUROPEP-instrument validation were presented at the EGPRN Meeting in Edirne-Turkey, 2015 (11).

### Participants

The study participants were selected using a three-stage random sampling. Initially, the country's region was selected, followed by general practices and patient selection. Twenty-five primary care practices (3.5%) from all five districts of the South-Central Region in Bulgaria were selected. The National Health Insurance Fund contract partners list was used, with random number assignment and selection, using a step-wise approach. The patient sample size was calculated at a maximum variance of 50% with 95% confidence interval and bounded to a maximum error of 5%. The sample size was set at 385 patients. Based on the literature review, the response rate of patients when mailing method is used ranges from 30% to 60% (12,13). The final sample was calculated at about 1000 participants. Eligible participants were aged 18 years or more and had valid health insurance. All of them had visited the primary care practice at least once in the preceding 12 months.

### Method

Initially, a telephone contact was established with the selected GPs. They were familiarized with the study objectives and consented to participate. The envelopes containing the EUROPEP questionnaire, instructions, informed consent forms, and an addressed and stamped envelope were delivered to the GP practices personally by the investigators or by using courier services. GPs handed out the envelopes to all eligible patients, in consecutive order, at the end of patient visit. In the instructions, the patients were asked to send the completed questionnaires to the investigator (RD) directly. A maximum of 40 adult patients per practice were consecutively included from those who had visited the practice for a consultation. A total of 1000 adult patients were invited to participate in the study. Written informed consent was obtained from all participants after the explanation of the study protocol.

To minimize the influence of physicians and bias when completing the questionnaire, the patients were instructed to complete the questionnaire at home and mail it to the research center, using the prepaid envelope. No personal identification was used and data sets contained only anonymous data. Therefore, the use of reminders or assessment of non-response bias was not possible. Unique questionnaire numbers ensured the correct identification of each general practice and allowed for the comparison of general practice characteristics with patients’ evaluation. Data were collected and analyzed at the research center.

### Statistical analysis

Descriptive statistic parameters (mean,±standard deviation [SD]) and percentages were calculated. Internal consistency was assessed using Cronbach’s alpha and average inter-item correlation. We defined an alpha of 0.80 as the lowest acceptable value (6,14). For the evaluation of intra-rater reliability, the split-half method was used and Spearman-Brown coefficient was calculated (rsb). An average inter-item correlation of at least 0.50 was regarded as good (14,15). Intra-class correlation coefficient (ICC), using the test-retest method, was also used to estimate the inter-rater reliability to assess consistency and reproducibility. The Wilcoxon signed-rank test was applied to compare item scale scores obtained during the test and re-test.

The item-scale correlation coefficients were calculated to determine whether each item on a scale was substantially related to the total score computed from the other items on that scale.

Construct validity was assessed by correlations of scale scores and evaluating the relationship between patient satisfaction and nine additional questions included in the questionnaire, similarly to the Norwegian and Portugues studies (3,16). The results obtained from each patient during test re-test were inputted, cleaned, cross checked, and analyzed with the corresponding patient’s previous results. Data were processed by IBM SPSS Statistics 22 software. The level of statistical significance was set at *P* < 0.05.

For each question, we calculated the percentage of responders, considering an item response rate of 90%-100% as good, 80%-90% as acceptable, and <80% as problematic (17).

According to the revised EUROPEP-2006 instrument and the user's manual, we accepted a benchmark of scale’s scores of 75% or above, ie, the percentage of positive patient evaluations of general practice care (4 or 5 on the Likert scale), corresponding to “good” and “excellent” rates to each item (8).

Exploratory factor analysis with the principal axis factoring extraction method was used to assess the underlying structure of the items and orthogonal rotation, using the Varimax method in the Final Bulgarian Version (3,16). Initially, sampling adequacy was assessed by using the Keiser-Meyer Olkin test (KMO) and the Bartlett's test of sphericity. Severely violated assumption of multivariate normality distribution of the data excludes the application of confirmatory factor analysis.

## RESULTS

### Psychometric quality of Bulgarian EUROPEP-instrument

*Internal consistency and test-retest reliability.* The item-scale correlation coefficient for all items is satisfactory (r >0.70). The overall Cronbach’s alpha for Bulgarian EUROPEP-instrument is 0.958 (for “clinical behaviour” is 0.95 and for “organisation of care” 0.81). Additionally, the internal consistency in each of the 5 subscales is considered satisfactory (Table 1). The high reliability of the instrument was confirmed by split-half method (0.96) and ICC-coefficient (0.97). Positive scores on “clinical behavior” dimension were significantly related only to positive scores on perceived health status (r_sb_ = 0.23, *P* = 0.004), however, this correlation was almost negligible. The sex and age of the respondents did not influence their evaluations of GPs.

It was found that Chronbah’s alpha coefficient between the question 12 and question 17 was very high (0.985), which allowed us to combine the two questions (Table 1). Also, during the cognitive interviews, patients argued that there was a conceptual and construct equivalence between Q12 and Q17. Taking into account this opinion, the construct and the meaning of Q12 was integrated with that of Q17. The resulting Q16 reads: “Explaining the purpose of the medical check and preparing for what to expect from other specialists, hospital care, tests and treatments” (Table 2).

**Table 2:** Results of construct validity for the Bulgarian EUROPEP-questionniare obtained by factor analysis, with factors and factor loadings (sorted by weight of coefficients)*

Perceived patient evaluations of general practice care	Factor 1	Factor 2
Q13. Helping you deal with emotional problems related to your health status?	0.826	
Q9. Thoroughness?	0.822	
Q14. Helping you understand the importance of following his or her advice?	0.822	
Q15. Knowing what s/he had done or told you during consultation?	0.814	
Q10. Physical examination of you?	0.812	
Q12. Telling you what you wanted to know about your symptoms and/or illness?	0.793	
Q2. Interest in your personal situation?	0.791	
Q16. Explaining the purpose of the medical check and preparing for what to expect from other specialists, hospital care, tests and treatments?	0.783	
Q7. Quick relief of your symptoms?	0.781	
Q8. Helping you to feel well so that you can perform your normal daily activities?	0.758	
Q3. Making it easy for you to tell him or her about your problem?	0.750	
Q11. Offering you services for preventing diseases (eg, health checks, tests, immunizations and etc.)	0.740	
Q1. Making you feel you had time during consultation?	0.732	
Q4. Involving you in decisions about your medical care?	0.731	
Q5. Listening to you?	0.713	
Q6. Keeping your records and data confidential?	0.662	
Q20. Being able to speak to the general practitioner on the telephone?		0.831
Q19. Getting through to the practice on the telephone?		0.823
Q22. Providing quick services for urgent health problems?		0.769
Q18. Getting an appointment to suit you?		0.736
Q21. Waiting time in the waiting room?		0.572
Q17. The helpfulness of the staff (other than the doctor)? % of Variance after Rotation: Varimax with Kaiser Normalization	48.50	0.569 28.49

*Principal axis factoring method.

Several ammendments were introduced to the final version of the questionnaire. All statements were converted to questions and a true zero was added, including answers (eg, “I do not have an opinion”). Also, verbal statements were included alongside with the numeric values of the scales (verbal numeric rating scale).

*Construct validity*. To confirm the construct validity of the Bulgarian EUROPEP-questionniare, exploratory factor analysis was performed by using the principal axis factoring extraction method with pairwise deletion of missing values revealed evidence for a 2-factor structure related to perceived patient evaluations of general practice care (Table 2). All items were organized into two subscales, “clinical behavior” (16 items) and “organization of care” (6 items). The KMO test and the Bartlett’s test of sphericity showed that the data were adequate for factorial analysis (KMO = 0.971 and Bartlett’s test *P* < 0.001). The two factors with eigenvalues >1 accounted for 77.0% of the total variation in these two items: factor 1 explained 72.9% of the total variation and factor 2 explained 4.1%. The high values of factor loading (>0.700) of each of the items in factor 1 required the use of rotation. After the rotation analysis, factor 1 explained 48.5% of the variance and factor 2 explained 28.5%. The level of factor-loadings for all items was >0.6. Factor analysis with eigenvalues <1 broke the first factor “clinical behavior” into two almost equal sub-factors - items 9-16 and items 1-8. However, it did not reach the five different domains of EUROPEP questionnaire.

The construct validity testing was assessed through correlations of scales and comparisons of responses to some additional questions included in the questionnaire (Table 3). Both “clinical behavior” and “organization of care” scales correlated significantly with general health status and number of GP consultations over the previous year. However, the low coefficient values indicated no significant relationship. Our study confirmed that a better health status is associated with more positive evaluation of care (χ^2^ = 56.08, *P* = 0.005).

**Table 3:** Correlation between scale scores and other variables (n = 496)

Scale /Item	Organization of care		Clinical behavior	
	Spearman’s correlation coefficient	*P* (two-tailed)	Spearman’s correlation coefficient	*P* (two-tailed)
Perceived health status	0.141	0.004	0.110	0.029
Number of visits to the GP	0.295	0.001	0.246	0.001

### Cross-sectional study using the Bulgarian EUROPEP-instrument

#### Patients’ and GPs’ characteristics

Of a total of 511 completed and returned questionnaires, 15 (2.9%) were discarded due to incomplete information (missing data were more than 50%) and 496 were finally processed (overall item-response rate of 49.6%). The mean±SD age of respondents was 53.4 ± 15.2 years (Table 4).

**Table 4:** Characteristics of the patients (n = 496) who completed the EUROPEP questionnaire

Patients’ characteristics	No. (%) of patients*
Gender	
women	180 (36.8)
men	309 (63.2)
Residence	
urban	398 (81.7)
rural	89 (18.3)
Level of education	
low	52 (10.6)
medium	249 (50.9)
high	188 (38.5)
Ethnicity distribution	
Bulgarian	447 (91.2)
Turkish	37 (7.6)
Roma	2 (0.4)
other	4 (0.8)
undisclosed	6 (1.2)
Employment status	
students	13 (2.7)
employed	264 (53.8)
pensioners	186 (38.0)
unemployed	27 (5.5)
Number of visits to the GP practice in the last 12 months (Mean)	
one	60 (12.2)
more than one	234 (47.7)
monthly	197 (40.1)
Perceived health status	
very good/excellent	90 (18.6)
good	239 (49.5)
neither good / nor bad	133 (27.6)
fair/poor	18 (3.7)
very fair/poor	3 (0.6)
Presence of chronic disease	
yes	267 (54.5)
no	223 (45.5)

*Percentages are calculated after excluding missing answers.

With respect to GP practices, 24 (96%) were solo GP practices, and 18 (72%) GPs were women. Comparison between the sample structure and the general population regarding the type of GP practice revealed no statistically significant differences (χ^2^ = 0.443, *P* = 0.505).

#### Ceiling effect and item response rate to Bulgarian EUROPEP-instrument

The item response rate was high with a small number of missing answers (Table 5). All items except item 21 had a ceiling effect larger than 50% (range: 50.6%-66.3%). For all items, the distribution was skewed to ‘excellent’. The most positive evaluations of general practice care (4 or 5 on the Likert scale) were 80.5% for all 22-items. Confidentiality of medical records (68.2% ‘excellent’ vs 0.4% ‘very poor’) and the GPs listening skills (67.1% ‘excellent’ vs 0.9% ‘very poor’) were most appreciated by patients. On the other hand, “Waiting times in the waiting room” and “Booking a convenient appointment” were the items rated most poorly. The benchmarking of the positive answers (the gold standard) was achieved in 21 items.

**Table 5:** Descriptive statistics, ceiling effect, and percentages of patients’ evaluations of the items for the Bulgarian EUROPEP-instument*

Bulgarian EUROPEP items	No. (%) of answers missing	Mean (SD)	No. (%) of not applicable/ not relevant answers^†^	Excellent (Ceiling effect)	Very good	Good	Fair	Poor
1. Making you feel you had time during consultation?	2 (0.4)	4.35 (0.96)	9 (1.8)	60.2	22.1	11.3	4.9	1.4
2. Interest in your personal situation?	1 (0.2)	4.36 (0.97)	3 (0.6)	61.8	21.1	10.0	5.7	1.4
3. Making it easy for you to tell him or her about your problem?	4 (0.8)	4.36 (0.99)	7 (1.4)	62.7	19.4	11.3	4.5	2.1
4. Involving you in decisions about your medical care?	1 (0.2)	4.30 (0.99)	18 (3.6)	57.7	24.7	9.6	6.1	1.9
5. Listening to you?	3 (0.6)	4.49 (0.84)	3 (0.6)	67.1	18.8	10.6	2.9	0.9
6. Keeping your records and data confidential?	3 (0.6)	4.55 (0.75)	18 (3.6)	68.2	20.6	9.5	1.3	0.4
7. Quick relief of your symptoms?	1 (0.2)	4.33 (0.95)	7 (1.4)	59.0	21.1	15.2	3.3	1.4
8. Helping you to feel well so that you can perform your normal daily activities?	3 (0.6)	4.33 (0.93)	7 (1.4)	57.6	23.9	14.0	2.9	1.6
9. Thoroughness?	1 (0.2)	4.33 (0.99)	8 (1.6)	60.6	21.1	11.5	4.5	2.3
10. Physical examination of you?	3 (0.6)	4.38 (0.97)	2 (0.4)	62.9	19.6	11.6	4.1	1.8
11. Offering you services for preventing diseases (eg, health checks, tests, immunizations and etc.)	11 (2.4)	4.34 (1.01)	7 (1.4)	60.8	22.2	10.3	3.7	3.1
12. Telling you what you wanted to know about your symptoms and/or illness?	2 (0.4)	4.31 (0.99)	9 (1.8)	58.4	23.1	10.1	4.5	3.9
13. Helping you deal with emotional problems related to your health status?	2 (0.4)	4.18 (1.07)	16 (3.2)	58.4	23.3	11.1	5.6	1.6
14. Helping you understand the importance of following his or her advice?	2 (0.4)	4.26 (1.06)	10 (2.0)	53.3	23.6	14.2	5.6	3.1
15. Knowing what s/he had done or told you during consultation?	1 (0.2)	4.31 (1.04)	24 (4.8)	58.1	21.5	12.4	4.8	3.3
16. Explaining the purpose of the medical check and preparing for what to expect from other specialists, hospital care, tests and treatments?	1 (0.2)	4.27 (1.07)	9 (1.8)	61.4	19.1	11.7	5.1	2.8
17. The helpfulness of the staff (other than the doctor)?	9 (1.8)	4.27 (0.95)	13 (2.6)	53.6	27.0	13.1	5.3	1.1
18. Getting an appointment to suit you?	9 (1.8)	4.18 (1.06)	11 (2.2)	52.7	23.5	15.8	4.8	3.2
19. Getting through to the practice on the telephone?	11 (2.2)	4.32 (1.03)	9 (1.8)	60.7	21.0	10.7	4.6	2.9
20. Being able to speak to the general practitioner on the telephone?	11 (2.2)	4.39 (0.99)	6 (1.2)	64.5	19.0	10.0	4.0	2.5
21. Waiting time in the waiting room?	7 (1.4)	3.65 (1.25)	4 (0.8)	33.8	22.7	26.4	9.1	8.0
22. Providing quick services for urgent health problems?	7 (1.4)	4.28 (1.05)	29 (5.8)	59.8	18.7	13.3	6.1	2.2

*Percentages are calculated from the total number of patients included in the study (n = 496), excluding the “missing” and “not relevant” answers.

†One of the possible answers was “I can not answer”.

## DISCUSSION

The validation process of current study revealed a satisfactory level for Cronbach’s alpha and Spearman coefficients and identified some areas requiring improvement in general practice. Compared to the results of other studies, the calculated internal consistency coefficient (Cronbach’α) of the aggregated scores for all five sub-scales was very high (6,12,13,16,18-20). It was also found out that the item-scale correlation exceeded the value of 0.70 for all items in the sub-scales, whereas in other studies, the respective values were over 0.40 (Italy) and over 0.50 (Norway) (16,20).

Recent studies reveal similar data, taking into consideration the mailing of the questionnaires (12,13,21). A survey in England with a very large sample (nearly two million respondents) reported suboptimal response rates (40.6%) (22). In a study in Slovenia, the response rate was about 84% (12). The response rates in 16 European countries varied from 47.1% to 89.0% (6).

The current study reviewed that the most evaluations of general practice care are 4 or 5 on the Likert scale for all of the EUROPEP questions. Similar to our results is the percentage of patients in Slovenia. Their positive evaluations (good/excellent) were 80.0% or higher for all items, except for the waiting time (18,23).

In our opinion, mean scores and overall high patient evaluations are satisfactory. In comparison, in Turkey the mean percentage rate of satisfaction was calculated at 88.3% (24). The mean scores and ceiling effect are consistent with previous EUROPEP studies (12,16-18,20). Similarly to the Slovenian study, our results showed that the items “keeping your records and data confidential” and “listening to you” were the most highly rated (over 88.8% and over 85.9% ‘excellent’ or ‘good’ rates), respectively (12). Most Bulgarians as well, rate highly the option to receive GP's medical advice on the phone and the availability of prophylactic and preventive services. These findings are broadly consistent with other literature data on patient evaluations of their care (6,18). Unlike the results of our study, Akturk et al (13) the established highest positive ratings for the items “providing quick services for urgent health problems”.

Similar to other studies, our results, indicate that patients were less satisfied with “organization of care” compared to “doctor-patient relationships and “clinical behavior” (12,18,22,25). Our results show that waiting time in the waiting room was the item rated most poorly, similar to the ratings provided in Germany (26). It seems that in Bulgaria and in Turkey, in terms of organization of care, waiting time in the waiting room is the least satisfactory item (18,27,28). Interestingly, Pakistani patients give highest ratings for “listening to you”, whereas “waiting time” was rated most poorly (25). Unlike our results, participants from the above-mentioned study, consider “respecting patient confidentiality” as unimportant (25). Interesting results were established in 16 other countries, participating in an international study - the percentage of good and excellent scores for “keeping confidentiality of medical records” was very high (6,23). Our results are comparable to the results from the multicenter study carried out in several European countries (Table 6) (23).

**Table 6:** A comparison of the results of patient evaluations of general practice care in Bulgaria and in eight other European countries

Bulgarian EUROPEP items	Percentage of patients with answers 4 or 5 on five-point Likert scale		
	from Bulgarian study	from eight European countries included in Slovenian study (6)	
		range	average for all eight countries
Making you feel you had time during consultations	82.3	(87.4-95.1)	89.6
Interest in your personal situation	82.9	(77.1-95.2)	87.9
Making it easy for you to tell him or her about your problem	82.1	(85.1-93.9)	89.2
Involving you in decisions about your medical care	82.4	(83.2-93.7)	86.9
Listening to you	85.9	(88.0-95.3)	91.6
Keeping your records and data confidential?	88.8	(91.2-97.0)	94.7
Quick relief of your symptoms?	80.1	(75.3-92.8)	86.5
Helping you to feel well so that you can perform your normal daily activities?	81.5	(83.4-93.6)	88.5
Thoroughness?	81.7	(84.8-94.4)	89.8
Physical examination of you?	82.5	(82.4-94.4)	88.9
Offering you services for preventing diseases (eg, health checks, tests, immunizations and etc.)	83.0	(79.9-90.3)	86.7
Telling you what you wanted to know about your symptoms and/or illness?	81.5	(83.3-96.2)	89.1
Helping you deal with emotional problems related to your health status?	81.7	(72.6-91.1)	83.2
Helping you understand the importance of following his or her advice?	76.9	(82.1-93.1)	87.3
Knowing what s/he had done or told you during consultation?	79.6	(78.3-91.2)	85.9
Explaining the purpose of the medical check and preparing for what to expect from other specialists, hospital care, tests and treatments?	80.5	not applicable*	not applicable*
The helpfulness of the staff (other than the doctor)?	80.6	(83.8-94.6)	89.9
Getting an appointment to suit you?	76.2	(76.0-97.4)	88.6
Getting through to the practice on the telephone?	81.7	(65.4-95.6)	86.3
Being able to speak to the general practitioner on the telephone?	83.5	(68.6-94.3)	82.7
Waiting time in the waiting room?	56.5	(63.9-82.9)	72.1
Providing quick services for urgent health problems?	78.5	(84.0-98.0)	91.7

*Not applicable because of combining the two questions: “Explaining the purpose of tests and treatments” and “Preparing what to expect from specialists”.

All items of the current study had skewed frequency distributions, suggesting a ceiling effect. This fact indicates that the large majority of patients had positive experiences. It might be speculated that our results reflect cultural traits and/or specifics in the organization of care – in fact, likewise, in many other countries, people are reluctant to express negative ratings even if they reflect their actual feelings (23,29). In our study, it was found that patients’ satisfaction was not affected by the type of the practices. The satisfaction ratings tended to increase with lower number of GPs in the practice as shown in a study from Switzerland (30). It is debatable, whether the cultural bias in the EUROPEP-instrument is able to delineate the differences, noted between the countries (31). We believe that further research on cultural validation will contribute to better knowledge of the specificity of patient evaluations in Bulgaria. We also take into consideration the options for improving the instrument itself.

Patients in Denmark were more satisfied with male GPs, whereas Bulgarian patients felt more satisfied and emotionally supported by their female GPs (6). Furthermore, the results from another study did not differentiate between the type of practice (solo or group) and patient’s preferences (18). Unfortunately, we were not able to explore the practice size as a predictor of satisfaction. Patients in the UK were more satisfied if they were able to “get through on telephone” if the practices were located in rural areas. Differently, our study did not find such correlation. On the contrary, patients in Germany were more satisfied if practices were located in urban areas (29).

As established in other studies healthier patients give better evaluations of health services (20,31). German patients with a self-reported chronic condition generally report higher satisfaction, but this is not the case with all aspects of care (32).

We also found that patients who visit the general practice more often were generally more satisfied with their GP, similar to patients in Slovenia (6,33).

Our cross-sectional study had several limitations. The overall response rate was unsatisfactory. After the linguistic validation process and the cross-cultural adaptation, the Bulgarian EUROPEP-instrument included 22-items. In fact, minimum amendments were made to the original EUROPE-instrument - the Bulgarian version is shorter by one question.

Representativeness could not be claimed regardless of the random sampling method, used as patients in the current study were mainly recruited in the cities. Overall, the sample is not representative for the general popultion. Unfortunately, comparative analysis between responders and non-responders could not be accomplished due to missing data.

The response rate to the survey questions was low; therefore, the results might be affected by response bias. The mailing of the questionnaires as well as the non- use of reminders are likely explanations to it. According to the study protocol, GPs were asked to recruit patients in a consecutive order, starting at a random point of time, however, no feed back was received to check the actual recruitment process.

Based on this, it is assumed that results for overall patient evaluation and comparability are skewed. Therefore, more additions and revisions should be made to the Bulgarian EUROPEP-questionnaire based on the conceptual equivalence and cultural relevance of the content of the questionnaire for the Bulgarian population. This could be achieved through expert discussions and patient’s cognitive debriefing. The larger-scale testing will be subject of another detailed publication.

In conclusion, the present study identified two scales in the Bulgarian EUROPEP-instument with satisfactory psychometric properties. However, Bulgarian cultural, economic, and health system characteristics and the established high ceiling effects indicate the need for further instrument development and future research. Additional research is needed to further clarify patients’ evaluations of care in general, and in terms of specific aspects, in order to answer how they reflect optimal care and outcomes. The results of the representative study will provide information necessary to better management and political decisions making. The result will be improved quality of general medical care in our country, as well as providing baseline data for international comparisons.

## References

[1] Van Royen P, Rees CE, Groenewegen P (2014). Patient-centred interprofessional collaboration in primary care: challenges for clinical, educational and health services research. An EGPRN keynote paper.. Eur J Gen Pract.

[2] Crow R, Gage H, Hampson S, Hart J, Kimber A, Storey L (2002). The measurement of satisfaction with healthcare: implications for practice from a systematic review of the literature.. Health Technol Assess.

[3] Roque H, Veloso A, Ferreira PL (2016). Portuguese version of the EUROPEP questionnaire: contributions to the psychometric validation.. Rev Saude Publica.

[4] Baker R (1996). Characteristics of practices, general practitioners and patients related to levels of patients’ satisfaction with consultations.. Br J Gen Pract.

[5] Baker R, Streatfield J (1995). What type of general practice do patients prefer? Exploration of practice characteristics influencing patient satisfaction.. Br J Gen Pract.

[6] Wensing M, Mainz J, Grol R (2000). A standardised instrument for patient evaluations of general practice care in Europe.. Eur J Gen Pract.

[7] Grol R, Wensing M, Mainz J, Jung HP, Ferreira P, Hearnshaw H (2000). European Task Force on Patient Evaluations of General Practice Care (EUROPEP) Patients in Europe evaluate general practice care: an international comparison.. Br J Gen Pract.

[8] EUROPEP. 2006, Revised Europep instrument and user manual, Center for Quality of Care Research, Michel Weinsing (co-ordinator). Available from: http://www.equip.ch/files/25/europep_2006rapport.pdf. Accessed: November 19, 2015.

[9] Beaton DE, Bombardier C, Guillemin F, Ferraz MB (2000). Guidelines for the process of cross-cultural adaptation of self-report measures.. Spine.

[10] Beatty P, Willis G (2007). Research synthesis: the practice of cognitive interviewing.. Public Opin Q.

[11] Dimova R, Asenova R, Torniova B, Doikov I. Validation of the Bulgarian version of EUROPEP-EUROPEP-instrument for patients’ evaluations of general practice care - preliminary results. Abstract book EGPRN 2015; 57. Available from: http://meeting.egprn.org/images/call_prog/EGPRN%20Edirne-Turkey2015.pdf. Accessed: May 25, 2016.

[12] Kersnik J (2000). An evaluation of patient satisfaction with family practice care in Slovenia.. Int J Qual Health Care.

[13] Akturk Z, Dagdeviren N, Sahin EM, Ozer C, Yaman H, Goktas O (2002). Patients evaluate physicians: the EUROPEP instrument.. Journal of 9 Eylul University Medical Faculty.

[14] Sitzia J (1999). How valid and reliable are patient satisfaction data? An analysis of 195 studies.. Int J Qual Health Care.

[15] Streiner DL, Norman GR. Health measurement scales. A practical guide to their development and use. New York: Oxford University Press; 2003.

[16] Bjertnaes OA, Lyngstad I, Malterud K, Garratt A (2011). The Norwegian EUROPEP questionnaire for patient evaluation of general practice: data quality, reliability and construct validity.. Fam Pract.

[17] Grol R, Mainz J, Wensing M (2000). A standardised instrument for patient evaluations of general practice care in Europe.. Eur J Gen Pract.

[18] Dağdeviren N, Akturk Z (2004). An evaluation of patient satisfaction in Turkey with the EUROPEP instrument.. Yonsei Med J.

[19] Vedsted P, Sokolowski I, Heje HN (2008). Data quality and confirmatory factor analysis of the Danish EUROPEP questionnaire on patient evaluation of general practice.. Scand J Prim Health Care.

[20] Milano M, Mola E, Collecchia G, Del Carlo A, Giancane R, Visentin G (2007). Validation of the Italian version of the EUROPEP instrument for patient evaluation of general practice care.. Eur J Gen Pract.

[21] Pit SW, Vo T, Pyakurel S (2014). The effectiveness of recruitment strategies on general practitioner's survey response rates - a systematic review.. BMC Med Res Methodol.

[22] Kontopantelis E, Roland M, Reeves D (2010). Patient experience of access to primary care: identification of predictors in a national patient survey.. BMC Fam Pract.

[23] Petek D, Künzi B, Kersnik J, Szecsenyi J, Wensing M (2011). Patients’ evaluations of European general practice–revisited after 11 years.. Int J Qual Health Care.

[24] Aktürk Z, Ateşoğlu D, Çiftçi E (2015). Patient satisfaction with family practice in Turkey: Three-year trend from 2010 to 2012.. Eur J Gen Pract.

[25] Ali NS, Khuwaja AK, Kausar S, Nanji K (2012). Patients’ evaluations of family practice care and attributes of a good family physician.. Qual Prim Care.

[26] Klingenberg A, Bahrs O, Szecsenyi J (1999). How do patients evaluate general practice? German results from the European Project on Patient Evaluation of General Practice Care (EUROPEP).. Z Arztl Fortbild Qualitatssich.

[27] Dimova R. Quality of general practice care – measurement, evaluation and management: doctor theses. Medical University of Plovdiv; 2003.

[28] Velikova-Zlatanova R, Zlatanova T. Patient satisfaction with GPs as a method for qality assessment. In: 2-nd Conference of the Association of General Practice/Family Medicine of Southeast Europe. Plovdiv: Collection of Reports; 2011.

[29] Wensing M, Vedsted P, Kersnik J, Peersman W, Klingenberg A, Hearnshaw H (2002). Patient satisfaction with availability of general practice: an international comparison.. Int J Qual Health Care.

[30] Sebo P, Herrmann FR, Bovier P, Haller DM (2015). What are patients’ expectations about the organization of their primary care physicians’ practices?. BMC Health Serv Res.

[31] Heje HN, Vedsted P, Sokolowski I, Olesen F (2008). Patient characteristics associated with differences in patients’ evaluation of their general practitioner.. BMC Health Serv Res.

[32] Goetz K, Campbell S, Rochon J, Klingenberg A, Szecsenyi J (2011). How do chronically ill patients evaluate their medical care? An observational study with 46919 patients in 676 primary care practices of direct relevance to person-centered medicine.. Int J Pers Cent Med.

[33] Kersnik J, Svab I, Vegnuti M (2001). Frequent attenders in general practice: quality of life, patient satisfaction, use of medical services and GP characteristics.. Scand J Prim Health Care.

